# Preventing invasive meningococcal disease in early infancy

**DOI:** 10.1080/21645515.2021.1979846

**Published:** 2022-04-28

**Authors:** Jessica Presa, Lidia Serra, Catherine Weil-Olivier, Laura York

**Affiliations:** a Vaccine Medical, Development, Scientific, and Clinical Affairs, Pfizer Inc, Collegeville, PA, USA; b Global Vaccines Medical Development and Scientific, and Clinical Affairs, Pfizer Inc, Collegeville, PA, USA; c Honorary Professor of Pediatrics, University Paris 7, Neuilly-sur-Seine, France; d York Biologics Consulting LLC, Wayne, PA, USA

**Keywords:** Infant, maternal vaccination, meningococcal, passive immunity, vaccination

## Abstract

This review considers the pathogenesis, diagnosis, and epidemiology of invasive meningococcal disease in infants, to examine and critique meningococcal disease prevention in this population through vaccination. High rates of meningococcal disease and poor outcomes, particularly for very young infants, highlight the importance of meningococcal vaccination in early infancy. Although effective and safe meningococcal vaccines are available for use from 6 weeks of age, they are not recommended globally. Emerging real-world data from the increased incorporation of these vaccines within immunization programs inform recommendations regarding effectiveness, appropriate vaccination schedule, possible long-term safety effects, and persistence of antibody responses. Importantly, to protect infants from IMD, national vaccination recommendations should be consistent with available data regarding vaccine safety, effectiveness, and disease risk.

## Introduction

*Neisseria meningitidis* is an obligate human pathogen that colonizes the respiratory tract and typically results in asymptomatic carriage.^1^^,2^ For reasons not fully understood, the bacteria can pass into the bloodstream and cross the blood–brain barrier, resulting in septicemia and meningitis, respectively, phenomena known as invasive meningococcal disease (IMD). Although generally uncommon, IMD onset is sudden and unpredictable and is still associated with high case fatality rates (CFRs; i.e., 6.9%–12.8% in a recent meta-analysis) and significant morbidity among survivors (20% or more), including limb amputation and neurologic deficits.^1,3–5^ Infants (i.e., <12 months of age) represent a vulnerable population at greater risk of IMD and adverse outcomes compared with other age groups.^4^ As initial clinical findings in infants are frequently nonspecific, the diagnosis and management of IMD in this cohort can be especially challenging.^6^

The incidence of IMD in any given country is variable because of temporal, geographic, and serogroup fluctuations as well as because of the emergence of hypervirulent clones from different serogroups.^7,8^ Epidemiologic studies indicate that of the five most common disease-causing meningococcal serogroups (i.e., A, B, C, W, and Y), serogroup B is prevalent in many parts of the Americas, Australasia, Europe, and North Africa; serogroup C disease occurs frequently in some countries within South America, Asia, and Africa.^9^ Additionally, a hypervirulent strain of meningococcal serogroup W has emerged recently in several regions, such as South America, Europe, Australasia, and sub-Saharan Africa, with observed differences in risk, clinical presentation, and outcomes of affected age groups.^10^

Various factors are thought to contribute to the increased risk of IMD in infants. The immaturity of the immune system makes this population more susceptible to infections, and risk factors associated with IMD in general are also applicable to the increased risk in infants, including close contact with an infected individual, crowded living conditions, and exposure to smoke or viral infections.^11^ Although transplacentally acquired maternal antibodies (i.e., those generated by the mother from prior colonization or exposure to bacteria with cross-reactive antigens) may provide protection in some young infants, protective levels wane within the first few months of life because these antibodies are catabolized over time.^6,12,13^ Thus, effective strategies are needed to protect infants against IMD.

Several vaccines protecting against disease caused by serogroups A, B, C, W, and Y are approved for use in infants. Factors determining if and how a country incorporates meningococcal vaccination commonly include IMD burden by serogroup, clinical outcomes, cost-effectiveness of the strategy, and national health priorities; aspects such as equity, budget impact, societal preference, and peace-of-mind benefit may also play a role.^14^ Although quantitative and comprehensive cost-benefit analysis of IMD vaccination is not considered in the current review, this is undoubtedly a critical aspect weighed in the decision-making process. It is consensus expert opinion that vaccine cost-effectiveness evaluations are complicated by the unpredictability of IMD and by inconsistencies associated with estimating both the indirect costs of the disease (e.g., productivity loss, premature death, inability to work, additional education or welfare needs, sequelae) and the indirect benefits of vaccination.^14^ Of note, although several recent cost-effectiveness analyses of serogroup B vaccines have fallen outside accepted quality-adjusted life-year thresholds, there is concern that the standard methods used may not fully account for vaccine impact.^14^ However, despite the heightened susceptibility of infants to IMD and the availability of safe and effective meningococcal vaccines, universal immunization strategies have not yet been widely implemented in this age group.^15^

This review examines meningococcal disease prevention in infants through vaccination, while also considering the potential of transferred maternal antibodies to provide additional protection in this population. IMD pathogenesis, diagnosis, and epidemiology in infants will first be briefly discussed.

## Pathogenesis and diagnosis of invasive meningococcal disease in infants

Clinical aspects of IMD in infants highlight the difficulties in achieving a definitive diagnosis, which is necessary for prompt and appropriate treatment that can minimize poor outcomes.^16^ Notably, signs and symptoms of IMD in infants are typically nonspecific and often consistent with initial symptoms of a febrile, nonserious viral infection, which often allows the disease to progress rapidly without treatment before being identified as an invasive bacterial infection.^6^ IMD progresses rapidly among all pediatric patients, with an estimated median time between onset and hospital admission of 13 hours compared with 20 to 22 hours in those 5 to 16 years of age.^5^ Early symptoms in infants (i.e., those occurring 0–4 hours from onset) include fever and irritability, followed by poor feeding, nausea/vomiting, coryza, and drowsiness. Rash in infants is reported to occur at an estimated median of 8 hours after onset. Late symptoms (i.e., those occurring 13–20 hours after onset) include photophobia, unconsciousness, bulging fontanel, stiff neck, seizure, and thirst. Additionally, infants experience a higher rate (e.g., 21% of children experienced ≥1 complication compared with 15% of adults in a population-based study) of long-term complications from IMD (in particular, hearing loss and seizures, as well as amputation and skin scarring) compared with older age groups (i.e., individuals 5 years and older),^17^ which is likely to result in long-term adverse effects on health.

According to the case definition from the European Union, confirmed IMD cases include patients meeting the laboratory criteria of either isolation of *N meningitidis* from a normally sterile site or purpuric skin lesion, detection of *N meningitidis* nucleic acid from a normally sterile site or purpuric skin lesion, detection of *N meningitidis* antigen in cerebrospinal fluid (CSF), or detection of gram-negative diplococci in CSF.^18^ A definitive diagnosis of IMD relies highly on CSF examination via lumbar puncture (LP). Given the invasiveness and inherent risks associated with the procedure in infants, particularly the very young, LPs may not be initially performed, which could lead to many missed diagnoses of meningitis.^16,19^ However, in European countries, it is the authors’ experience that LPs are commonly performed in infants for CSF examination concurrently with other diagnostic methods to initiate treatment rapidly.

The literature regarding the pathogenesis of IMD in neonates (i.e., infants <28 days of age) is even more limited compared with older infants. One study compared a 10-day-old neonate with meningococcal sepsis to literature reports spanning a 97-year period, which included 31 cases of IMD.^20^ In 13 cases, IMD developed in the first week of life, with typically insignificant clinical signs and symptoms; symptoms became distinctive approximately 1 week after onset and included irritability (53%), fever (47%), hypotension (31%), and petechiae (31%). Diagnosis was made by blood or CSF cultures, with the most common serogroups being serogroup B (28%) and serogroup C (19%). However, in 44% of cases, the serogroup could not be classified. Nearly one-third of the cases were fatal, with 50% of these occurring in patients who developed disease in the first week of life. Among survivors, significant morbidity was observed in four patients, including hydrocephalus, subdural empyema, and spinal dysfunction.

## Epidemiology of invasive meningococcal disease in infants

Worldwide, infants are generally at greater risk of IMD compared with other age groups.^4,21^ In countries with comprehensive surveillance systems (Table 1^22–25^), the incidence of IMD in infants ranges from 0.83 per 100,000 in the United States (2018^23^) to 23.1 per 100,000 in New Zealand (2017^25^) versus comparative incidences of 0.10 to 2.3 per 100,000 in the general population.^22–25^ In countries within the European Union/European Economic Area, IMD incidence rates in infants as high as 22.44 per 100,000 were reported in 2018 compared with rates of up to 1.82 per 100,000 in the general population.^24^ In these countries and regions, CFRs for IMD in infants range from 7.6% in Europe (2018^24^) to 12.9% in the United States (2018^23^); CFRs for IMD in the general population range from 8.0% in New Zealand (2017^25^) to 12.0% in the United States (2018^23^).^22–25^

**Table 1.** Epidemiology of invasive meningococcal disease in infants

Country/region (year)	Incidence in infants (per 100,000)	Incidence in general population (per 100,000)	Case fatality in infants, %	Case fatality in general population, %
The Americas				
Canada (2011)^22^	7.15	0.51	–	8.1
United States (2018)^23^	0.83	0.10	12.9^a^	12.0
European Union (2018)^24^	8.40	0.62	7.6	11.6
Croatia	16.45	0.76	–	–
Hungary	18.07	0.41	17.6	15.0
Ireland	19.30	1.82	22.2	19.1
Malta	22.44	0.84	–	–
Poland	11.41	0.52	6.7	11.6
Portugal	20.87	0.55	11.1	10.5
Slovakia	18.73	0.66	11.1	14.7
United Kingdom	14.31	1.17	4.4	8.8
Western Pacific				
New Zealand (2017)^25^	23.1	2.3	–	8.0

^a^Deaths with known outcome.

Notably, the available data are predominantly from Western regions, surveillance systems are not in place globally, and differences in medical practice make it difficult in many countries to obtain laboratory confirmation of meningococcal disease. As such, an underestimation of the true global burden of IMD in infants is likely, particularly in regions, such as Africa, which has a large burden of IMD in the general population.^21^

There is also a paucity of information regarding IMD epidemiology by month of age in infants. Data from the US Centers for Disease Control and Prevention suggest that variability may exist between older and younger infants in terms of disease incidence. Surveillance data for 2006–2012 in the United States estimated an average of 2.74 cases per 100,000 and 6 deaths each year of culture-confirmed IMD in infants, with the greatest risk in infants 2 to 5 months of age (3.34–3.45 annual cases per 100,000) and the lowest in older infants 10 to 11 months of age (1.54 annual cases per 100,000).^11^ Serogroup B disease was most commonly observed (1.76 annual cases per 100,000), followed by serogroup Y (0.54 annual cases per 100,000) and C (0.27 annual cases per 100,000) disease. These data are similar to a report of IMD epidemiology in England and Wales from 2006 to 2011, in which the number of IMD cases increased with each month of age from birth, peaking at 5 months of age (approximately 33 cases), and then generally decreasing thereafter (approximately 16 cases at 12 months of age).^26^ The vast majority of IMD cases in infants over this time period were serogroup B and MenB vaccines were not available at that time.

## Vaccination of infants against invasive meningococcal disease

### Considerations

Although around 50% of newborns may have naturally acquired anti-meningococcal bactericidal activity, this immunity decreases rapidly; by 6 to 12 months of age, few infants have serum bactericidal activity, and susceptibility to infection peaks.^27^ Therefore, prevention of IMD in infants through vaccination is critical to improve outcomes in this vulnerable population. The benefits of vaccination can be achieved both by direct vaccination of an individual and indirectly to the unvaccinated population.^28^ The latter encompasses herd protection, which reduces disease among those in the community who are not vaccinated, and importantly for very young infants, the transfer of maternal antibodies in utero.^6,28–31^

Although direct vaccination of an individual is often the most efficient means of protection against vaccine-preventable diseases, challenges exist in immunizing some populations, including the youngest infants. The greatest limitation of early infant vaccination is achieving sufficient immune responses because of the insufficiently developed innate and adaptive immune systems of neonates.^27,32^ In early infancy, Th1-type immunity and inflammasome pathways are dampened, leading to increased colonization of microbes and limited proinflammatory responses. These characteristics leave young infants susceptible to infection and minimize their ability to mount vaccine responses, particularly as this dampening directly coincides with increased risk of disease. This effect may be more pronounced for extremely or very preterm infants (i.e., <28 and 28–32 weeks of gestational age, respectively) as they do not benefit from the progressive increases in maternal antibody transfer to the fetus during pregnancy, which is preponderant in the third trimester.^33^ As such, only a very limited number of infant vaccine schedules begin before 6 weeks of age (e.g., oral polio vaccine, hepatitis B vaccine, Bacille Calmette-Guérin vaccine^15^), leaving infants at higher risk of other vaccine-preventable diseases in the first weeks or months of life.

A critical component of population-level protection is the effect of vaccination on the nasopharyngeal carriage of meningococci, which is a precursor for the development of IMD.^34^ The prevalence of meningococcal carriage in Western countries varies nonlinearly by age, with rates of 4.5% in infants, 7.7% in children 10 years of age, and peaking at 23.7% in 19-year-olds; the prevalence decreases thereafter (13.1% in those 30 years of age and 7.8% in those 50 years of age).^35^ In sub-Saharan Africa, meningococcal carriage is common in young children and remains high in teenagers and adults in some countries.^36^ Accordingly, vaccines and vaccination programs with high uptake and that reduce meningococcal carriage rates among adolescents and young adults have the greatest potential of affecting disease rates in other age groups, including infants.^34^

### Meningococcal vaccines and the impact of meningococcal vaccination programs

Vaccines are available for the prevention of meningococcal disease. The first widely used formulations contained meningococcal polysaccharides as vaccine antigens.^37^ These were formulated in plain polysaccharide bivalent (e.g., covering meningococcal serogroups A and C [MenAC]), trivalent (e.g., MenACW), and quadrivalent (e.g., MenACWY) formulations. However, meningococcal oligosaccharide antigens result in a weak humoral immune response in infants that is unable to confer protection against IMD for children younger than 2 years and thus are not recommended in this age group; the utility of these vaccines has predominantly been for control of localized outbreaks, including past serogroup A epidemics in sub-Saharan Africa.^37,38^

#### Meningococcal conjugate vaccines

Through conjugation of the meningococcal oligosaccharide to a carrier protein (e.g., to the tetanus toxoid [TT], diphtheria toxoid [DT], or nontoxic mutant of diphtheria toxin cross-reactive material 197 [CRM_197_]), induction of a T-cell–dependent response is achieved^39^ as demonstrated in infants from 6 weeks of age. Additional benefits of meningococcal conjugate vaccines include increased duration of protection and reduced nasopharyngeal carriage of the bacterium, the latter of which can at best interrupt transmission and thereby lead to herd protection.^39,40^ Currently available conjugate meningococcal vaccines include monovalent, bivalent, and quadrivalent formulations,^39^ with only a subset of the available vaccines licensed for use in young infants (Table 2). For instance, MenC conjugate vaccines using CRM_197_ (Menjugate [Novartis; Siena, Italy]) or TT (NeisVac-C [Pfizer Canada; Kirkland, Canada]) are available for infants.^42,43,50,51^ A combination TT conjugate vaccine targeting *Haemophilus influenzae* and meningococcal serogroup C disease (Menitorix [GlaxoSmithKline; Middlesex, UK]) is also available in some countries for use in those 2 months to 2 years of age.^49,50^

**Table 2.** Meningococcal vaccines licensed for use in infants

Vaccine	Description	Meningococcal serogroups (other antigens)	Licensed age range^a^
Polysaccharide conjugate (Monovalent)			
MenAfricVac^41^	TT conjugate	A	1–29 y
Menjugate^42^	CRM_197_ conjugate	C	≥2 mo
NeisVac-C^43^	TT conjugate	C	≥2 mo
Polysaccharide conjugate (Quadrivalent)			
Nimenrix^44^	TT conjugate	A, C, W, Y	≥6 wk
Menveo^45^	CRM_197_ conjugate	A, C, W, Y	≥2 mo
Menactra^46^	Polysaccharide diphtheria toxoid conjugate	A, C, W, Y	≥9 mo
OMV + polysaccharide			
VA-MENGOC-BC^47^	OMV (strain B:4:P1.19,15) + MenC polysaccharide	B, C	≥3 mo
Protein + OMV			
Bexsero (MenB-4 C)^48^	Recombinant protein + OMV (strain NZ98/254)	B	≥2 mo
Combination			
Menitorix^49^	TT conjugate	C (Hib)	2 mo–2 y

CRM_197_, cross-reactive material 197; Hib, *Haemophilus influenzae* type b; OMV, outer membrane vesicle; TT, tetanus toxoid.

^a^Licensed age range may vary by country.

Four MenACWY conjugate vaccines are currently licensed, of which three are approved for use in infants (Table 2). Two of them are licensed for use in infants in the United States, including a MenACWY conjugated to CRM_197_ (MenACWY-CRM_197_; Menveo [GlaxoSmithKline; Sovicille, Italy]), approved for use in infants as young as 2 months, and a MenACWY vaccine conjugated to DT (MenACWY-D; Menactra [Sanofi Pasteur Inc; Swiftwater, PA]), approved for use in a 2-dose schedule in infants from 9 months of age.^45,46^ MenACWY-CRM_197_ is administered as a 3-dose series in infants vaccinated at 2 months of age (at 2, 4, and 6 months) with a booster dose at 12 months; in infants and toddlers 7 to 23 months of age, a 2-dose series is used, with the second dose administered ≥3 months after the first and at ≥12 months of age.^45^ In the European Union, MenACWY-D is not licensed and MenACWY- CRM_197_ is approved, but in individuals from 2 years of age.^52^ A MenACWY vaccine conjugated to TT (MenACWY-TT; Nimenrix [Pfizer Ltd; Sandwich, UK]) is currently licensed in the European Union and other countries for use in infants as young as 6 weeks.^44,50^ MenACWY-TT is administered as a 2-dose primary series for infants between 6 weeks and 6 months of age, with a 2-month interval between doses; from 6 months of age, a single primary dose may be given.^44^ After completion of the primary MenACWY-TT schedule, a booster dose should be administered at 12 months of age.

A large body of evidence is available regarding the impact of the routine use of the MenC conjugate vaccine (MCCV; Table 3). An epidemiologic study from England and Wales from 1993/1994 to 2003/2004 considered the effect of the introduction of MCCV in 1999 into the routine infant immunization program at 2, 3, and 4 months of age, with a catch-up program offered to anyone younger than 18 years (extended to anyone younger than 25 years in 2001).^53^ In the overall population, the vaccination program had high uptake both in the targeted and catch-up populations, and resulted in a 93% decrease in serogroup C cases from 1998/1999 to 2003/2004. A systematic literature review found a reduction in MenC cases in England and Wales from 1993/2000 to 2000/2007 of 78%–87% in infants <1 year of age and 70%–98% in children 1–4 years of age.^54^ MCCV was also introduced in 2010 to the Brazilian vaccination schedule in children younger than 2 years, resulting in a progressive reduction of serogroup C IMD incidence in the overall population, from approximately 0.6 per 1 million in 2010 to 0.15 per 1 million in 2017.^55^ Likewise, the systematic literature review found a reduction in serogroup C cases of 67%–96% in children <2 years of age within 4 years of adding MCCV to the Brazilian vaccination schedule.^54^

**Table 3.** Overview of the effect of MCCV national immunization programs on serogroup C disease

Country	Vaccination program (year)	Effect on serogroup C disease
*MCCV included in infant NIP*		
England and Wales^53,54^	Routine infant NIP at 2, 3, 4 months of age and catch-up for <18 years of age (1999)	From 1998/1999 to 2003/2004: 93% decrease overall From 1993/2000 to 2000/2007: 78%–87% decrease in infants <1 year of age 70–98% decrease in children 1–4 years of age
Brazil^54,55^	Routine NIP in children <2 years of age (2010)	Incidence in the overall population: 0.6 per 1,000,000 (in 2010) 0.15 per 1,000,000 (in 2017) Within 4 years of MCCV inclusion in NIP: 67%–96% decrease in cases in children <2 years of age
*MCCV not included in infant NIP*		
The Netherlands^56^	1–18 years of age (2002)	Incidence before and after MCCV in directly vaccinated age groups: 1–5 years of age: 0.9–2.49 vs 0–0.69 per 100,000 6–14 years of age: 0.56–1.12 vs 0–0.06 per 100,000 15–18 years of age: 1.79–2.95 vs 0–0.64 per 100,000 Cases in populations not targeted for vaccination: 49% decrease in infants <1 year of age in first 3 months of the campaign vs same period in the previous year
Australia^57^	12 months of age and catch-up for those 1–19 years of age (2003)	Serogroup C incidence decreased in all age groups from before (2000–2002) to after (2010–2012) MCCV implementation, with the greatest effects in directly targeted populations: <1 year of age: 2.93 vs 0.34 per 100,000 1–4 years of age: 3.10 vs 0 per 100,000 5–14 years of age: 1.49 vs 0.04 per 100,000 15–24 years of age: 3.57 vs 0.07 per 100,000 All ages: 1.30 vs 0.06 per 100,000

MCCV, meningococcal serogroup C conjugate vaccine; NIP, national immunization program.

Variable indirect benefits of MCCV programs have also been reported in countries that did not include infants in their programs (Table 3). The Health Council of the Netherlands had recommended implementing MCCV as a 2-dose series at 5 and 6 months of age or as a single dose shortly after the child’s first birthday, with a large catch-up program up to 18 years of age.^58^ Because of the relatively low incidence of serogroup C disease in infants, vaccination after 1 year of age was considered an acceptable approach. With the introduction of the latter strategy in 2002 (i.e., introduction of 1 dose of MCCV to children and adolescents 1–18 years of age), the Netherlands Reference Laboratory for Bacterial Meningitis conducted a nationwide surveillance study to assess the direct and herd effects of MCCV.^56^ Comparing incidence before and after the introduction of MCCV in 2002, rates of serogroup C disease decreased among the age groups directly vaccinated (age 1–5 years, 0.90–2.49 per 100,000 vs 0–0.69 per 100,000, respectively; age 6–14 years, 0.56–1.12 per 100,000 vs 0–0.06 per 100,000; and age 15–18 years, 1.79–2.95 per 100,000 vs 0–0.64 per 100,000). Indirect benefits of MCCV were also seen in populations not targeted for vaccination; for instance, a 49% decrease in serogroup C cases was observed in infants <1 year of age during the first 3 months of the vaccination campaign compared with the same period in the prior year.

Australia implemented a national MCCV program in 2003 for children 12 months of age, with a staged catch-up program for those 1 to 19 years of age.^57^ IMD incidence decreased in all age groups after implementation of the program, with the greatest effect in serogroupC incidence in those age-based populations targeted for vaccination. Across all time periods, infants, who were not part of the program, had the highest IMD incidence, which was predominantly attributed to serogroup B disease. In infants, the average annualized incidence of serogroup C disease and non-serogroup C disease decreased by 89% and 60% after MCCV implementation, respectively; corresponding percentage decreases were 96% and 55% in the overall population. In all of these examples, very high uptake (>80%) of vaccines was quickly achieved in the cohorts targeted by the vaccination program.

The effect of meningococcal conjugate vaccines in reducing vaccine-type serogroup carriage has been reported, predominantly from studies associated with mass vaccination campaigns.^34^ For instance, in a study from the United Kingdom of meningococcal carriage of adolescents 15 to 19 years of age who were offered a single MCCV dose as part of the vaccination program,^59^ MCCV significantly decreased the prevalence of serogroup C carriage among vaccinated and unvaccinated individuals. In Chad, vaccination of people 1 to 29 years of age with a serogroup A meningococcal TT conjugate vaccine (PsA-TT; MenAfriVac [Serum Institute of India]) began in 2009 in selected regions, with an estimated 94% vaccine coverage in 2012.^60^ In regions that introduced PsA-TT, a decrease in meningococcal disease was accompanied by a dramatic decrease in serogroup A carriage in all age groups. However, the continued high incidence of serogroup A disease in nonvaccinated areas supports that transmission of the disease continued if serogroup A carriers remained. Collectively, these data suggest that the effect of meningococcal conjugate vaccines on carriage may affect transmission to, and consequently IMD rates among, unvaccinated populations, including potentially among unvaccinated infants.

#### Meningococcal outer membrane vesicle vaccines

MenB vaccines based on outer membrane vesicles (OMVs) have been used to control outbreaks of serogroup B disease in several countries including Cuba, Norway, France, and New Zealand.^61^ Importantly, because the OMV vaccine antigen is only from a single disease strain, OMV vaccines are predominantly effective against the homologous or clonal strain carrying the same porin protein A (PorA) as the vaccine.^62^

The first effective MenB OMV vaccine was developed and implemented in Cuba (VA-MENGOC-BC; Table 2). During a mass vaccination campaign commencing in 1989, VA-MENGOC-BC was administered to >3 million individuals 3 months to 24 years of age.^47^ Thereafter, the vaccine was added to the Cuban national immunization schedule as a 2-dose schedule administered at 3 and 5 months of age, with the aim of preventing new epidemic outbreaks; this schedule has been continuously maintained. Shortly after the development of VA-MENGOC-BC, the Norwegian Institute of Public Health developed MenBvac to address a serogroup B epidemic in Norway during 1988–1991 in children 13–16 years of age.^62,63^ Almost 20 years later, MenBvac was used in individuals <20 years of age to control an outbreak of a genetically similar strain in Normandy, France, from 2006 to 2012.^63^ In New Zealand, a heterologous serogroup B strain outbreak beginning in 1991 led to the development of the MeNZB vaccine, which was included in the national immunization program as a 3-dose schedule from 2004 to 2008, initially in individuals >6 months of age and then to those >6 weeks of age.^62,63^ In 2006, a fourth dose was added for infants 10 months of age because of observed waning immunity.^62^ More than 3 million doses were administered by the middle of 2006 to individuals 20 years and younger.^62,63^ The MenNZB vaccine was included as part of the routine infant vaccination schedule in New Zealand until 2008.^62^

The effectiveness of OMV vaccines appears to be considerably lower in infants and young children compared with older age groups, although this may be overcome to some extent by the use of multiple-dose schedules.^47,61^ For instance, a large decrease in IMD was observed in Cuba following the implementation of VA-MENGOC-BC, with incidence falling from a prevaccine peak of 14.3 per 100,000 in 1983 to 0.1 per 100,000 during 2008–2016.^61^ Associated vaccine effectiveness estimates range from 80% to 100% across all age groups; during 1997–2008, the mean effectiveness in infants was 84%.^47^ In Norway, a 29-month clinical trial estimated efficacy of VA-MENGOC-BC at 57% in 13–16 year olds.^62^ Importantly, an examination of IMD incidence in Cuba during the prevaccination period spanning from 1984 to 1988 showed a considerable decrease (from 14.1 to 8.8 per 100,000) before the introduction of the vaccine, thus making it challenging to decipher the true impact of the implementation of VA-MENGOC-BC.^64^ Similarly, a natural decline was observed in serogroup B disease in New Zealand (approximately 25 to 13 per 100,000) in 2001–2004 before the implementation of the MenNZB vaccine in 2004.^62^ An analysis of the disease incidence that differentiated the vaccine program effect from the natural decline reported that the decrease was accelerated after implementation of the vaccination program, suggesting an impact of the MenNZB vaccine. Moreover, vaccine effectiveness was estimated at 68%–77% over an average period of 3.2 years following the 3-dose series.

#### Meningococcal protein-based vaccines

More recently, protein-based vaccines were licensed for prevention of serogroup B disease, with the MenB-4C vaccine (Bexsero [GlaxoSmithKline Vaccines Srl; Siena, Italy]) indicated for use in infants from 2 months of age in Europe (Table 2).^6,48^ From 2 to 5 months of age, MenB-4C is administered on either a 3-dose or 2-dose primary schedule, whereas a 2-dose primary schedule is used for infants 6 to 11 months of age. A booster dose is required in both age groups.

Three years after implementation of MenB-4C into the infant immunization program in the United Kingdom, the incidence of serogroup B disease decreased by 75% among all children who were eligible for vaccination.^65^ In two Italian regions where MenB-4C has been included in the infant immunization program, the relative reduction in serogroup B disease was 65.4% at 4 years after implementation in Tuscany and 31.2% at 3 years after implementation in Veneto.^66^ In Portugal, where MenB-4C is not included in the national schedule, a case-control study of MenB-4C vaccination and serogroup B disease in children and adolescents indicated that 7.2% of patients with serogroup B disease were fully vaccinated versus 23.2% of matched controls, corresponding to an estimated effectiveness of 79%.^67^

Although direct effects of MenB-4C on disease rates have been realized, a corresponding effect on serogroup B carriage has not been demonstrated. In contrast to serogroup A and C conjugate vaccines, a large 2017–2018 Australian study of over 24,000 high-school students found that MenB-4C did not affect serogroup B carriage and therefore did not provide indirect protection (i.e., herd immunity).^68^ These findings are consistent with those from localized vaccination campaigns with MenB protein-based vaccines in association with university outbreaks or in Saguenay–Lac-Saint-Jean, Quebec, Canada, in which support for the effect of vaccination on meningococcal carriage and thus on herd protection was not found,^69–71^ and emphasize the need for direct protection (i.e., vaccination) against serogroup B disease for high-risk populations, including preschool children.^68^

### Recommendations for meningococcal vaccination of infants

A complex decision-making process precedes any alteration to a national immunization program. Factors that play a key role in determining the meningococcal vaccines implemented by individual countries include local IMD epidemiology (e.g., predominant serogroups and clinical disease burden) and economic aspects, such as cost-effectiveness and budgetary constraints.^14^ In addition, national health system priorities are usually a major consideration when contemplating any changes to an often crowded universal infant schedule, and in some countries societal expectation may also exert a strong influence.^72^ Reliable health-economic models are critical tools used in the decision-making process undertaken by vaccination advisory committees. However, the evaluation of meningococcal vaccine cost-effectiveness is particularly challenging, at least in part due to difficulties associated with accurately estimating the indirect benefits of vaccination.^14^ This is reflected in the changing position statements issued by the UK Joint Committee on Vaccination and Immunisation (JCVI) preceding the introduction of universal MenB-4C vaccination in 2015.^73^ On the basis of inadequate cost-effectiveness, an interim position statement issued by JCVI in 2013 recommended against introducing MenB-4C.^74^ This decision was widely debated in the public forum, and a subsequent model reevaluating cost-effectiveness took into account updated vaccine and disease burden data as well as indirect costs associated with litigation, care, and loss of quality of life for family members.^73,75^ Outcomes of this model indicated that infant vaccination with MenB-4C could be cost-effective if a discounted price was secured, and an ensuing position statement issued by JCVI recommended that MenB-4C should be implemented universally in infants at 2, 4, and 12 months of age.^75^ In some countries, meningococcal vaccines are licensed for use in infants but are not recommended or reimbursed,^76^ thus creating inequities and disparities in vaccination coverage based on family income.

Despite the availability of meningococcal vaccines for use in infants, only some countries now include recommendations regarding their use in this age group (Table 4).^15,73,77,78^ Several African countries recommend vaccination of infants with the MenA conjugate vaccine at 9 months of age, whereas countries in the Americas mostly recommend the MenACWY conjugate vaccine either as a single dose at 12 months or a 2-dose schedule at 3 and 5 months of age (with or without a booster dose).^15^ Numerous European countries recommend infant MenC conjugate vaccination, and a small number have also implemented a MenB 2- or 3-dose plus booster infant vaccine schedule. In France, a single-dose MenC vaccine at 12 months of age was initially recommended in 2010; however, because of insufficient vaccine coverage rates, the incidence of IMD in infants increased between 2010 and 2016 which led France to update the recommendation to a 2-dose schedule at 5 and 12 months of age in 2017.^82,83^ The Haute autorité de Santé very recently recommended the routine implementation of MenB vaccine for infants, but it is not yet reimbursed. Additionally, recent increases in serogroup B, C, and W cases among infants in Malta^84^ subsequently led to inclusion of MenACWY and MenB vaccines in the infant national immunization program.^76^ It is expected that within the coming years, MenACWY vaccines will replace MenC vaccines within infant vaccination programs, particularly in middle- and high-income countries to broaden the range of protection.^85^ Moreover, adolescent meningococcal vaccination, including primary vaccination and a booster dose, is already recommended in the United Kingdom and in some European member states.^76,86^ Overall, depending on country and vaccine, the earliest age that a meningococcal vaccine series is recommended to begin is at 2 months, knowing that some protection likely occurs after just one dose. The earliest age of completion of a multidose primary series, as is recommended for several meningococcal vaccines administered in infancy, occurs at 5 months, with a booster usually around 1 year of age.^15^

**Table 4.** Currently recommended meningococcal vaccination of infants.^15,73,77–81^

Region	Country	Vaccine	Schedule	Notes
Africa	Central African Republic	MenA conjugate	9 mo	
	Chad	MenA conjugate	9 mo	
	Gambia	MenA conjugate	12 mo	From April 2019; 12–24 mo
	Guinea	MenA conjugate	9–11 mo	From October 2020
	Ivory Coast	MenA conjugate	9 mo	
	Mali	MenA conjugate	9 mo	
	Nigeria	MenA conjugate	9 mo	From August 2019
	Niger	MenA conjugate	9 mo	
Americas	Argentina	MenACWY conjugate	3, 5, 15 mo	
	Brazil	MenC conjugate	3, 5, 12 mo	Reinforcement doses administered from 12 mo to 4 y
	Canada	MenC conjugate	12 mo	Given at 2 mo and 12 mo in British Columbia, Yukon, Northwest Territories; 4 mo and 12 mo in Alberta; 12 mo in Saskatchewan, Manitoba, Ontario, New Brunswick, Nova Scotia, Prince Edward Island, Newfoundland and Labrador, Nunavut; 12 mo in Quebec (if born before June 2019)
	Chile	MenACWY conjugate	12 mo	
	Colombia	MenACWY conjugate	2, 4, 6 mo	For outbreaks
	Cuba	MenBC	3, 5 mo	
	United States	MenACWY conjugate	2 mo	Travelers aged 2 mo–18 y to sub-Saharan Africa
Eastern Mediterranean	Libya	MenACWY conjugate	9, 12 mo	
	Saudi Arabia	MenACWY conjugate	9, 12 mo	
	Sudan	MenA	9 mo	
Europe	Andorra	MenB	2, 4, 13 mo	
		MenC conjugate	4, 15 mo	
	Cyprus	MenC conjugate	12–13 mo	
	France	MenC conjugate	5, 12 mo	
	Germany	MenC conjugate	12–23 mo	
	Greece	MenC conjugate	12 mo	
	Iceland	MenC conjugate	6, 8 mo	
	Ireland	MenB	2, 4, 12 mo	
		MenC conjugate	6, 13 mo	
	Italy	MenB	3, 4, 6, 13 mo or 7, 9, 13 mo	7/9/13 mo regimen for infants >6 mo of age
		MenC or MenACWY conjugate (by regional evaluation)	13–15 mo	MenC to MenACWY switch
	Lithuania	MenB	3, 5, 12–15 mo	
	Luxembourg	MenC conjugate	13 mo	
	Monaco	MenC conjugate	5, 12 mo	
	Portugal	MenC conjugate	12 mo	
	San Marino	MenACWY conjugate	13 mo	
		MenB	4, 6, 7, 13–14 mo	
	Spain	MenC conjugate	4, 12 mo	In 2 regions, MenACWY conjugate is administered instead of the second dose of MenC conjugate at 12 mo
	Switzerland	MenC conjugate	12 mo	
	United Kingdom	MenB	8, 16 wk, 1 y	
South-East Asia	Thailand	MenACWY conjugate	9 mo	
Western Pacific	Australia	MenACWY conjugate	12 mo	MenC to MenACWY switch
	China	MenA polysaccharide	6, 9 mo	

Men, meningococcal serogroup.

Data are current as of October 12, 2020.

## Discussion

In spite of well-established vaccination programs globally during childhood, infants and especially neonates still represent a vulnerable population for many infectious diseases, including IMD.^4,6,21^ The risk of IMD in infants persists despite increased disease awareness by physicians and parents or guardians, improved biological diagnosis through polymerase-chain reaction (PCR), early treatment, and effective antibiotic therapies without concerns of resistance. Therefore, effective routine immunization of infants against these diseases remains a global health priority.

Direct vaccination of infants against IMD is an important approach to protect this population against high mortality and long-term sequelae. Effective and safe meningococcal vaccines are available for use in infants as young as 6 weeks,^44,50^ although they are not used globally. Vaccine hesitancy regarding infant immunization schedules can decrease vaccine uptake and affect timely receipt of recommended immunizations within this population.^87^ Notably, the high rates of meningococcal disease and poor outcomes, particularly for very young infants,^4,11,17,21^ emphasize the importance of early use of meningococcal vaccination among this vulnerable population. With increased incorporation of meningococcal vaccines within infant immunization schedules, emerging real-world data should further inform recommendations regarding effectiveness, the optimal age and schedule for vaccination, and the persistence of antibody responses, as well as clarify any possible long-term effects on vaccine safety.

Importantly, to protect infants from IMD, national vaccination recommendations should be consistent with available data regarding vaccine safety and disease risk. Conjugate vaccine programs may be most impactful when a catch-up program in adolescents is also implemented and has high uptake, which will affect carriage and transmission in the community and bolster herd immunity.^34^ Although it is difficult to globalize IMD vaccinations for protection against serogroup B disease, the lack of evidence from well-powered studies of an effect of vaccination on carriage^68^ indicates that direct vaccination of at-risk populations, such as infants would be required.

In countries where meningococcal vaccination of infants is recommended, substantial differences exist in the recommended age for initiating vaccination. Vaccination programs begin as early as 2 months and as late as 12 to 13 months of age. Infant vaccination schedules should be considered in the context of the overall vaccination program across all age groups so as to consider the potential for population effects on infants from vaccination of older age groups (i.e., adolescents).

Based on the limited data available, very young infants (i.e., <2 months of age) are at high risk of IMD even compared with older infants.^11^ Given that very young infants cannot be directly vaccinated, other strategies to protect this population should be evaluated. For instance, direct vaccination of pregnant women (in conjunction with other preventive approaches) may provide immunity to infants against vaccine-preventable diseases during their first months of life.^6,88^ Maternally acquired antigen-specific immunoglobulin G (IgG), which is actively transferred across the placenta in utero, is the major contributor to immunity in early infancy.^6,29–31^ For women who have had prior antigen exposure before pregnancy (e.g., by past infection or vaccination), prenatal vaccination may also provide a booster response leading to high IgG responses to both the mother and the infant.^6,29^ Use of maternal vaccination to confer immunity to infants has been used in other infectious diseases,^89–92^ and data are emerging on meningococcal vaccination during pregnancy.^29,93–100^ Importantly, comprehensive data regarding the effectiveness, safety, and optimal timing of maternal vaccination against meningococcal disease, as well as regulatory approvals, are needed before such recommendations can be made.^6^ Such data might strengthen the evidence for the benefit of maternal vaccination to achieve passive infant immunity, including against IMD.

Vaccination programs worldwide are experiencing numerous challenges, including the dynamic nature of disease, vaccine hesitancy, and limited access of vulnerable populations to effective vaccines.^31^ Improvements in clinical and microbiological disease surveillance, such as the increased use of rapid tests, PCR, and molecular characterization, will allow more precise identification and evaluation of vaccination programs that reflect the dynamic nature of meningococcal disease. Improvements in the methodologies for disease surveillance are critical to ultimately evaluate the effectiveness of vaccines and vaccine recommendations through the accumulation of real-world data that is achieved with greater vaccine uptake. In the future, it is anticipated that approaches to improve the success of vaccination programs, including those in pregnancy and for direct vaccination of infants, will be better elucidated.
